# High-quality polycrystalline vanadium dioxide thin films deposited via pulsed laser deposition with high uniformity and consistency

**DOI:** 10.1007/s10854-025-15921-6

**Published:** 2025-10-11

**Authors:** Zhixiang Huang, Kyle Laskowski, Sai Rahul Sitaram, Eric Herrmann, S M Jahadun Nobi, Ke Ma, Xi Wang

**Affiliations:** Department of Materials Science and Engineering, College of Engineering, University of Delaware, Newark, DE 19716 USA

## Abstract

Vanadium dioxide (VO_2_) is a phase transition material that experiences significant shifts in electrical, optical, and mechanical properties near its transition temperature. Among various methods for depositing VO_2_ thin films, pulsed laser deposition (PLD) provides precise stoichiometric control, good versatility, and high consistency. In this work, we introduce an optimized PLD-based method for depositing high-quality polycrystalline VO_2_ thin films. Experimental results demonstrate a resistance change of over four orders of magnitude during the phase transition, accompanied by high uniformity, with a thickness variation of less than 2% across a 100 mm wafer, and reliable reproducibility over time.

**Supplementary Information:**

The online version contains supplementary material available at 10.1007/s10854-025-15921-6.

## Relevance summary

This work introduces an optimized pulsed laser deposition (PLD) approach for depositing high-quality polycrystalline vanadium dioxide (VO_2_) thin films. Experimental results demonstrate a resistance change exceeding four orders of magnitude across the phase transition, which is among the highest for reported polycrystalline VO_2_ thin films. Meanwhile, this approach provides exceptional uniformity, achieving a thickness variation of less than 2% over a 100 mm wafer. We have utilized this approach to deposit VO_2_ thin films for over a year, resulting in several samples for THz modulation applications. It has demonstrated high reproducibility over the past two years, even after several routine maintenance procedures. The large resistance changes across phase transition, exceptional uniformity, and high reproducibility confirmed the scalability and robustness of our method for large-scale applications. This work laid the foundation for integrating VO_2_ thin films into optical and electronic devices, paving the way for advanced applications that require precise control of material properties.

We believe our work makes a strong contribution in the following ways:

1. **Address a critical scalability challenge**: Although VO_2_ is widely recognized for its insulator-to-metal transition and its extensive functionality in optoelectronic and nanophotonic applications, practical implementation has been limited by the challenge of achieving uniform, high-quality thin films over large areas.
2. **Introduce a novel deposition strategy**: We present an optimized pulsed laser deposition (PLD) approach that incorporates a novel laser sweeping technique. This method improves film uniformity across a four-inch substrate and enables reproducible fabrication over extended periods.
3. **Demonstrate enhanced material performance for device applications**: The resulting VO_2_ thin films exhibit excellent device-relevant performance, such as sharp phase transitions, large resistance contrasts, and exceptional wafer-scale uniformity.
4. **Align with the multidisciplinary scope of Journal of Materials Science: Materials in Electronics**: This work is highly relevant to current research trends in reconfigurable nanophotonics, smart coatings, energy applications, and neuromorphic computing, and aligns well with the scope of *Journal of Materials Science: Materials in Electronics* to study “the synthesis, growth and processing of new materials.” We believe our manuscript will attract broad interest from readers in the fields of photonics, materials science, applied physics, and applied engineering.

## Introduction

Vanadium dioxide (VO_2_) has attracted significant research interest due to its insulator-to-metal transition (IMT) occurring near room temperature (68 °C), which is accompanied by a structural phase transition from a monoclinic insulating phase to a tetragonal metallic phase [1]. This concurrent electronic and crystalline transition leads to significant changes in electrical conductivity and optical properties, making VO_2_ highly attractive for applications in optoelectronics, thermal management, and nanophotonics [2–4]. The sharp contrast in its optical properties across the IMT has positioned VO_2_ as a promising candidate for dynamically reconfigurable optical devices, including light modulators [5], optical switches [6], and tunable metasurfaces [7].

VO_2_ has various applications, notably in smart windows, where its passive thermochromic properties enable the dynamic regulation of infrared transmission. This helps maintain indoor temperatures without requiring external power. As a result, VO_2_-based coatings are increasingly being developed for sustainable building applications [8]. Furthermore, its rapid phase transition and tunable optical properties have facilitated its integration into nanophotonic platforms, enabling advanced functionalities such as active beam steering [9], phase modulation [10], and infrared camouflage [11].

While extensive research has been conducted on VO_2_ in various forms, including single crystals, nanowires, and nanoflakes, these structures often face challenges related to scalability, reproducibility, and large-area integration [12]. These limitations have motivated increasing interest in high-quality VO_2_ thin films, which offer superior structural consistency, large-scale uniformity, and greater feasibility for practical device applications [13, 14]. As a result, the focus of recent research has shifted toward optimizing the synthesis, characterization, and integration of VO_2_ thin films to meet the growing demand for dynamic optical and electronic materials.

Multiple deposition techniques have been developed for fabricating high-quality VO_2_ thin films, each offering distinct advantages in terms of precision, scalability, and material control [12, 15]. Molecular beam epitaxy (MBE) is a widely used technique that enables the deposition of VO_2_ with exceptional purity and atomic-level precision, making it highly suitable for fundamental studies and applications requiring defect-free films [16, 17]. Chemical vapor deposition (CVD) and atomic layer deposition (ALD) offer excellent conformality and scalability, enabling uniform coatings over complex surfaces, which benefits large-area device fabrication [18]. Sputtering techniques, such as radio frequency (RF) magnetron sputtering, offer a cost-effective approach with tunable deposition parameters, facilitating industrial-scale production [19, 20].

Among these methods, pulsed laser deposition (PLD) stands out for its ability to produce high-quality VO_2_ thin films with precise stoichiometric control [21]. PLD operates by directing high-energy laser pulses onto a target in a controlled gas environment, resulting in the rapid ablation of the target material and the formation of a plasma plume. The ejected species then condense onto the substrate, forming a thin film [22, 23]. One of the primary advantages of PLD is its ability to precisely control the stoichiometry of the deposited film, ensuring high compositional fidelity [13, 22]. Additionally, PLD allows for the fine-tuning of film properties by adjusting parameters such as laser fluence, repetition rate, oxygen partial pressure, and substrate temperature, thereby enabling control over crystallinity, phase transition behavior, and defect density [24, 25]. However, challenges related to large-area uniformity, phase transition consistency, and reproducibility still remain, preventing further advancements in deposition methods that would improve the reliability and scalability of VO_2_-based devices.

To overcome these challenges, we introduce an improved PLD-based deposition method that uses a new laser sweeping technique to achieve greater uniformity over a four-inch substrate. This method boosts film consistency, increases resistance change during phase transition, and maintains reproducibility over long deposition periods, thereby advancing the potential of VO_2_ thin films for practical optoelectronic and nanophotonic applications.

## Method

The PLD system is highly versatile because various deposition parameters can be precisely controlled, including oxygen (O_2_) pressure, O_2_ flow rate, deposition temperature, substrate-target distance, laser pulse repetition rate, laser pulse energy, and the number of laser pulses. Collectively, these parameters form a complex system that requires examining each factor and its role in the final thin film.

**Fig. 1:**
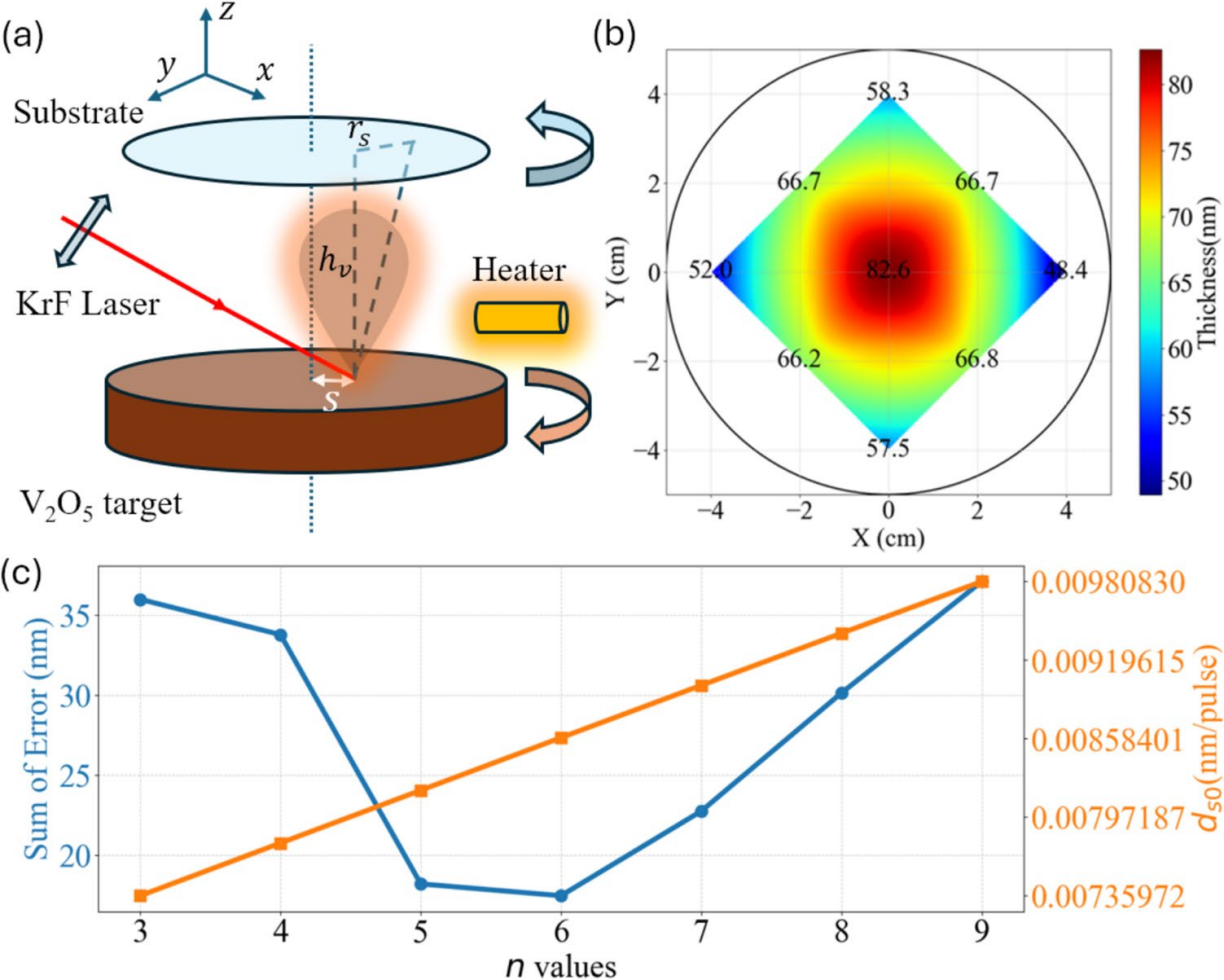
**a** Schematic of the material transfer process in the PLD system. **b** Ellipsometry measured the thickness of the calibration VO_2_/Sapphire wafer. **c** Left y-axis: sum of error between the simulation and the measured thickness values with different *n* values. Right y-axis: required *d*_*s0*_ values for the simulation to match the measured center thickness

The primary attribute we prioritize is the uniformity of the thin film. Due to the multi-objective nature of the optimization problem, we developed a reality-based simulation model that mimics the deposition process in the PLD chamber. Since thin film materials are ablated and transferred onto the substrate each time a laser pulse hits the target, we can break down the entire process, involving thousands of laser pulses, and start by analyzing the effect of a single pulse. The schematic diagram of the material transfer process in the PLD system is shown in Fig. 1a. Many researchers have created models that describe this point source material transfer process [26]. Our model is based on one of the first models that described this process, proposed by Scheller et al. [27],

$$\frac{{d}_{s}}{{d}_{s0}}=\frac{1}{{\left[{1+\left(\frac{{r}_{s}}{{h}_{v}}\right)}^{2}\right]}^{\frac{n+3}{2}}}$$

where ${d}_{s}$ is the local film thickness, ${d}_{s0}$ is the film thickness right on top of the point materials source, ${r}_{s}$ is the distance on the substrate from the point of maximum thickness, ${h}_{v}$ is the height of the substrate over the materials source, and $n$ is the parameter that depicts the cross-sectional profiles of the vapor plume of the evaporated materials. With a larger $n$ value, the profile is more elliptical, while with a smaller $n$ value, the profile is more spherical.

With this model, we initially performed multiple verification depositions, followed by multi-point ellipsometry measurements on the substrate to retrieve the thickness, as shown in Fig. 1b. In our model, the *d*_*s*0_ represents the thickness of VO_2_ deposited in the center of the substrate with each laser pulse hitting the target. The total thickness difference at each location between the calculated value and the experimental value is the sum of error, measured in nm. We found that the value $n=6$ matches our experimental results best, as shown in Fig. 1c. Throughout multiple calibration depositions, we enhanced the accuracy and robustness of our model, gradually narrowing the gap between simulated results and actual deposition. Ultimately, we developed an ideal model that offers reliable and precise predictions for the deposited VO_2_ thin film. Details are provided in the Supporting Information. With the verified model, we used ${r}_{s}=90 mm$ and a specifically designed laser raster strategy to deposit our VO_2_ thin film.

After successfully verifying our VO_2_ deposition model, our next goal was to optimize the quality of the deposited VO_2_ thin film. To quantify the quality, we measured its resistance values at 30 and 90 °C. This resistance ratio (R30 °C/R90 °C ratio) between the two temperatures is the key quantitative indicator we use during this optimization. Throughout the process, we kept the previously optimized parameters unchanged to ensure uniform deposition. These unchanged parameters include the laser raster logic (details in the Supporting Information), the number of laser pulses (66,000 pulses), the laser repetition rate (23 Hz), the laser energy (400 mJ/pulse), the distance from the substrate to the target (90 mm), and the rotational speeds of the substrate and target (13 and 17 rpm).

Apart from these unchanged parameters, several other factors required optimization, including the deposition and annealing temperature as well as the O_2_ pressure. To investigate their effects, we first analyzed the effect of O_2_ pressure while keeping other deposition conditions unchanged. Based on previous studies on VO_2_ deposition using PLD, we initially adopted an O_2_ pressure of 10 mTorr and iterated through other pressures to compare the resulting quality of the VO_2_ thin film. Our findings indicated that an O_2_ pressure of 10 mTorr during deposition yields the highest quality VO_2_ thin films, aligning with other literature [28]. Further details are provided in the Supporting Information, with Figure S4 illustrating the morphology of the VO_2_ thin film deposited at different O_2_ pressures. Once the optimal O_2_ condition was identified, we examined how changing deposition and annealing temperatures affected the quality of the VO_2_ film. As reported in previous studies, the annealing temperature strongly affects the final morphology and phase composition of VO_2_ structures [2, 29, 30]. As explained in the Supporting Information, Figure S5 shows that a temperature of 600 °C is necessary to produce high-quality VO_2_ polycrystalline thin films.

## Results

**Fig. 2:**
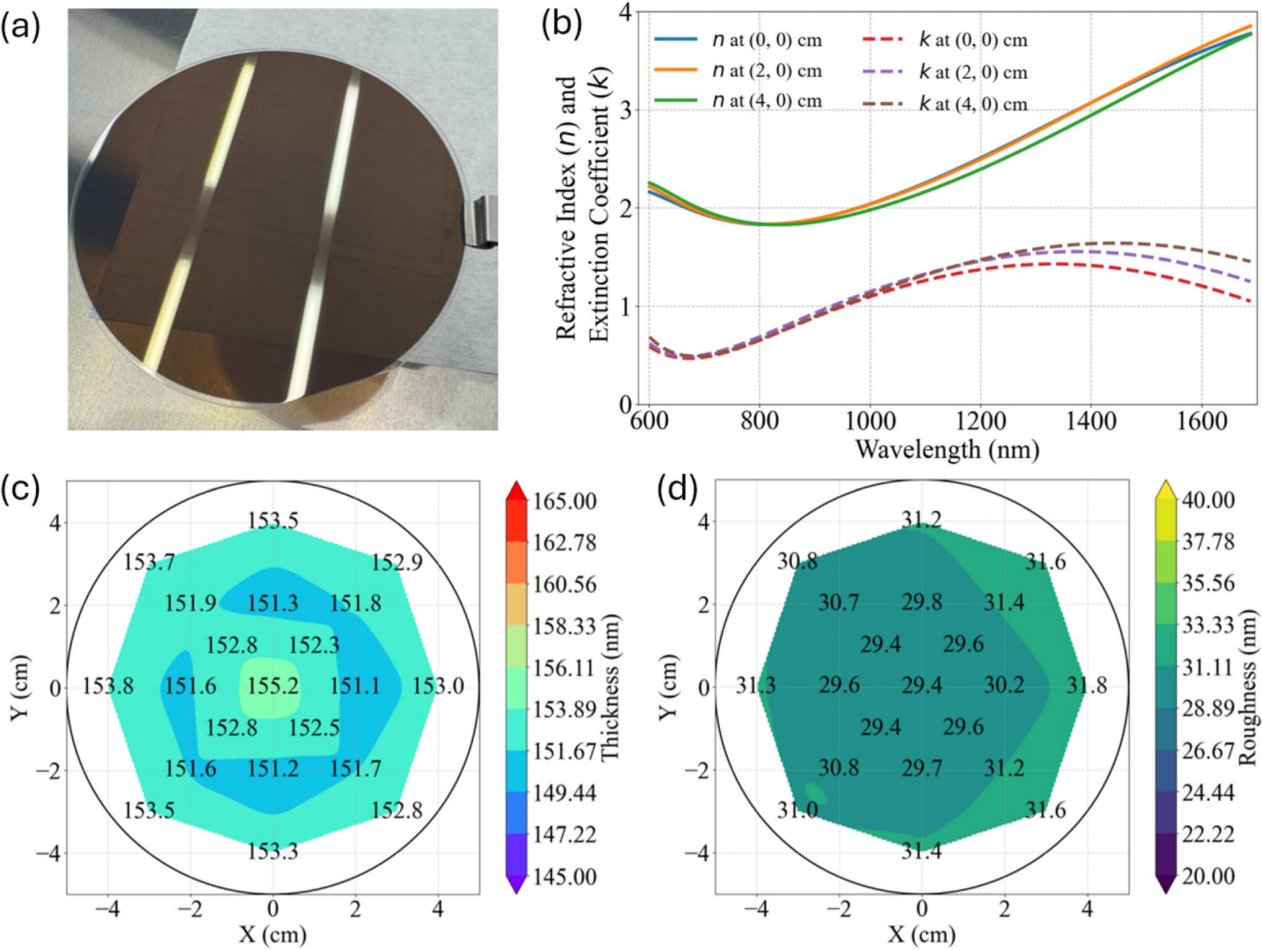
**a** A picture of the VO_2_/Sapphire wafer. **b** Fitted refractive index *n* and extinction coefficient *k* values at different locations on the wafer by ellipsometry. **c** Fitted thickness distribution of the VO_2_/Sapphire wafer by ellipsometry. **d** Fitted roughness distribution of the VO_2_/Sapphire wafer by ellipsometry

Using the optimized PLD method, we successfully fabricated VO_2_ thin films on a 4-inch sapphire substrate, as shown in the optical image in Fig. 2a. To evaluate the uniformity and quality of the films, we performed ellipsometry measurements at 21 points on the 4-inch sapphire wafer, as detailed in the Supporting Information. The fitted optical parameters as a function of wavelength at different locations on the VO_2_/Sapphire wafer are shown in Fig. 2b. Using these fitted parameters, the thickness values in Fig. 2c indicate a variation of less than ± 2%, demonstrating high uniformity across the entire 4-inch wafer. In addition, the fitted roughness values in Fig. 2d also show consistent uniformity across the wafer. These roughness values are comparable to the RMS roughness of 29.4 nm measured by atomic force microscopy, as shown in the Supporting Information. This high level of uniformity highlights the effectiveness of our innovative sweeping technique in controlling film deposition. In the past, the PLD method was well-known for maintaining uniformity within a small area [12, 31]. The thickness uniformity of our sample is comparable to the VO_2_ film (1.2%) grown on a 3-inch sapphire wafer by MBE [32], which is complex and costly. Wafer-scale uniform VO_2_ films have also been demonstrated by sputtering [33–35] and water–vapor oxidant methods [36] on 2-inch wafers. However, the uniformity of our sample is superior on a 4-inch wafer, providing better scalability.

**Fig. 3:**
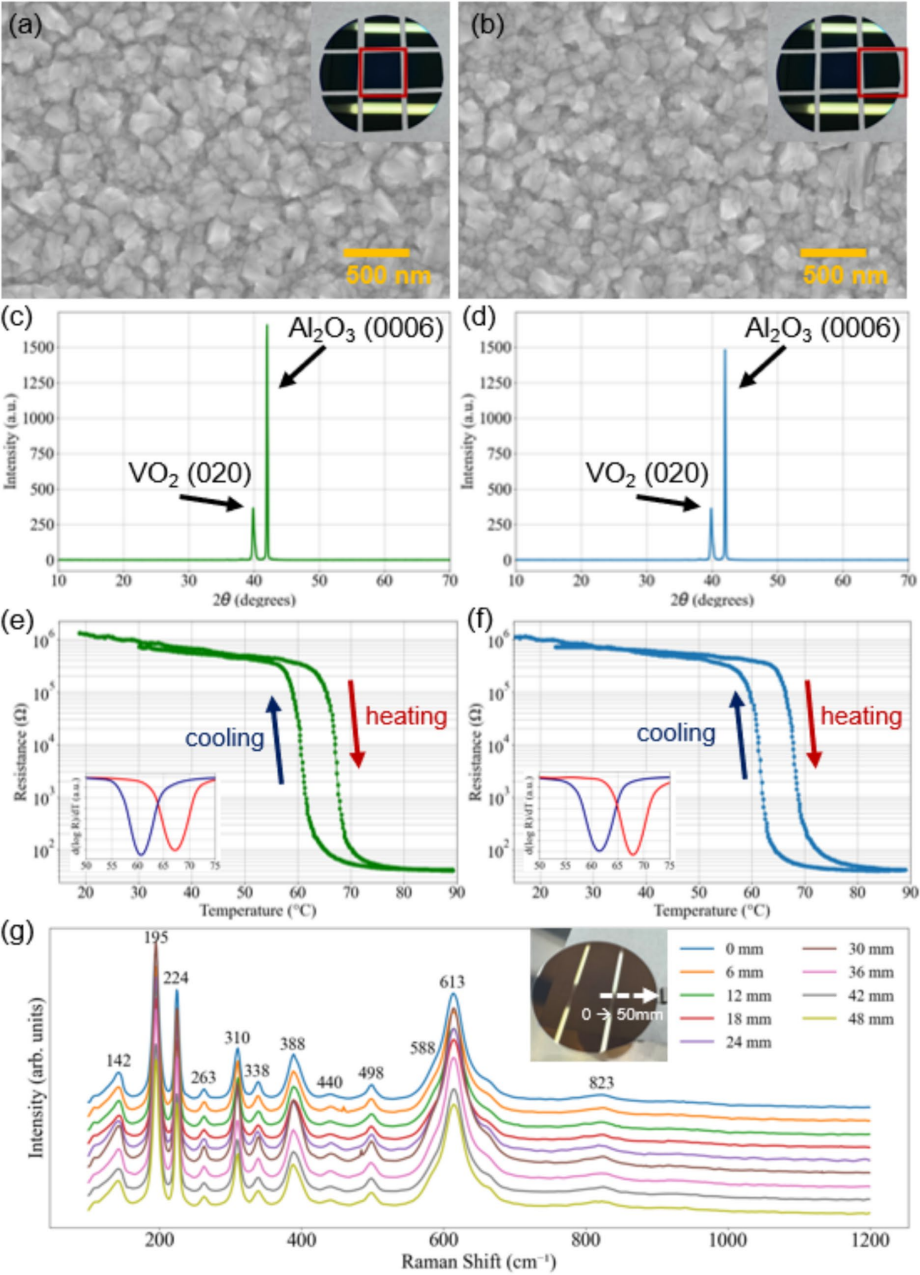
SEM images taken **a** at the center and **b** near the edge (at [4 cm,0] point) of the VO_2_/Sapphire wafer. Insets: Optical image of the cleaved wafer. Red squares indicate the piece used for the SEM images. XRD spectra taken **c** at the center and **d** near the edge (at [4 cm, 0] point) of the VO_2_/Sapphire wafer. Results of R–T measurements taken **e** at the center and **f** near the edge (at [4 cm, 0] point) of the VO_2_/Sapphire wafer. Insets show the differential d(log*R*)/d(*T*) as a function of temperature curves for heating (red) and cooling (blue) branches. **g** Raman Scattering in the VO_2_ film deposited on a sapphire substrate. The measurements begin at the edge of the wafer and progress inward by increments of 6 mm (color figure online)

Additional characterization of the VO₂ thin films was carried out using Scanning Electron Microscopy (SEM) and X-ray Diffraction (XRD) to examine the morphology and crystallinity of the VO_2_ thin film. Figure 3a and b shows SEM images taken from two different locations on the wafer, with Fig. 3a from the center and Fig. 3b near the edge. The consistent morphology seen in both images indicates the uniformity of the deposition process.

Figure 3c and d shows the XRD spectra from the same sample locations as the SEM images. The diffraction patterns confirm the presence of distinct peaks characteristic of VO_2_, indicating successful formation of the monoclinic phases. The strong peak at approximately 40° corresponds to the VO_2_(020) lattice plane, while the peak at around 42° originates from the sapphire substrate’s (0006) plane [37]. This may be due to the growth of monoclinic VO_2_ films textured in the direction (002) or (020) [38]. The phase transition behavior of VO_2_ thin films was evaluated through resistance–temperature (R–T) measurements. The film exhibited a significant change in resistance of over four orders of magnitude during the insulator-to-metal transition. Figure 3e and f shows the R–T measurement results taken at the center and the edge of the wafer. At the center, the resistance changes from 908 kΩ (30 °C) to 40 Ω (90 °C). During heating and cooling, a hysteresis loop of 6.5 °C is observed. At the edge, the resistance changes from 721 kΩ (30 °C) to 42 Ω (90 °C). During heating and cooling, a hysteresis loop of 6.4 °C is observed. The consistently high R30 °C/R90 °C ratio across the wafer highlights the excellent phase transition properties achieved with our optimized deposition parameters. The summary of R30 °C/R90 °C ratio compared to other literature is listed in Table S1 in the Supporting Information. The transition temperature aligned with reported values for VO_2_, occurring near 68 °C, and demonstrated sharp switching behavior, further confirming the film’s high quality. Figure 3g shows Raman spectra of the VO_2_ film measured from the wafer edge to the center in 6 mm steps. All characteristic peaks of VO_2_ (e.g., 142, 195, 224, 263, 310, 338, 388, 440, 498, 588, 613, and 823 cm^−1^) are clearly observed. No peaks corresponding to V_2_O_3_ or V_2_O_5_ are detected, indicating phase purity and good stoichiometry of the VO_2_ film [39].

**Fig. 4:**
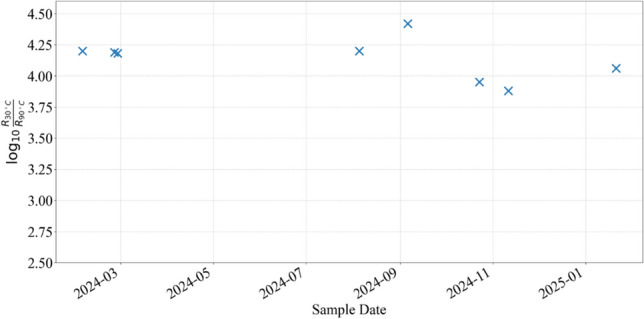
The magnitude of resistance changes of VO_2_ thin films deposited throughout the time

Finally, to evaluate the consistency of our process, we conducted R-T measurements on multiple VO_2_/sapphire samples produced over an extended period, beginning in 2024, as shown in Fig. 4. All samples demonstrated stable phase transition behavior, characterized by a high R30 °C/R90 °C ratio and minimal variation, which confirms the reliability and reproducibility of our PLD method. These results establish our method as a robust approach for producing VO_2_ thin films with high optical and electrical properties suitable for large-scale applications.

## Conclusion

In this study, we demonstrated a novel and optimized PLD method for fabricating high-quality VO_2_ thin films with high uniformity and phase transition properties. By systematically optimizing deposition parameters and introducing a sweeping technique, we achieved a polycrystalline VO_2_ thin film with an exceptional R30 °C/R90 °C ratio exceeding four orders of magnitude change in resistance during phase transition, along with uniform thickness variations of less than 2% across a 100 mm sapphire wafer. The uniformity and reproducibility achieved across multiple samples confirmed the scalability and robustness of our method for large-scale applications. This work laid the foundation for integrating VO_2_ thin films into optical and electronic devices, paving the way for advanced applications that require precise control of material properties.

## Supplementary Information

Below is the link to the electronic supplementary material.

Supplementary file1 (PDF 1037 KB)

## Data Availability

The data that support the findings of this study are available from the corresponding author upon reasonable request.
