# Supplementary material for: High-quality polycrystalline vanadium dioxide thin films deposited via pulsed laser deposition with high uniformity and consistency
Source: J Mater Sci Mater Electron. 2025 Oct 11;36(29):1850. doi: 10.1007/s10854-025-15921-6 (PMC12515231; doi:10.1007/s10854-025-15921-6)
Supplement: Supplementary file 1 — Supplementary file1 (PDF 1037 KB) [file 10854_2025_15921_MOESM1_ESM.pdf]

Supporting Information

High-Quality Polycrystalline Vanadium Dioxide Thin Films Deposited via Pulsed Laser Deposition with High Uniformity and Consistency

Zhixiang Huang, Kyle Laskowski, Sai Rahul Sitaram, Eric Herrmann, S M Jahadun Nobi, Ke Ma, and Xi Wang

Department of Materials Science and Engineering, College of Engineering, University of Delaware, Newark, DE 19716, USA

Corresponding author:
Xi Wang – Email: wangxi@udel.edu

Instruments and materials details

All our samples are fabricated in the University of Delaware Nanofabrication Facility (UDNF) cleanroom. The pulsed laser deposition (PLD) system is a PLD-4000 system manufactured by the PVD Products[1]. Figure S1 shows the inside of the PLD chamber.

The PLD system is equipped with a Coherent COMPex Pro 205 KrF excimer laser system. The wavelength of the laser is 248 nm. The angle of incidence of the laser on the target is 60°. The optics system includes a beam-forming aperture with X-Y slits that clip the edge of the raw beam coming out of the laser, a high-quality focus lens AR-coated on both sides that is used for laser beam focusing, a kinematic mirror which includes a programmable linear actuator that provides the ability to raster the laser beam across the target surface, etc.

The PLD system handles a single wafer up to 4” (100mm). And it has a target carousel with three target pedestals to handle up to three 5” diameter targets or up to six 2” targets. The target we use is a V_2O_5 target of 5” (125mm) in diameter, 1/4" in thickness, and 99.9% in purity. A combination usage of different targets is feasible but not discussed within the scope of this paper. The distance between the substrate and the target is controllable between 90 mm and 140 mm. Here, we used a 90 mm distance for all our depositions because it provides the fastest deposition growth rate.

Heaters protected inside quartz tubes are placed in the PLD chamber, in the middle of the target and substrate. The chamber temperature is measured by a type K thermocouple, which is inserted through an opening in the top of the heater. This temperature reader is not a direct indication of the substrate temperature since the thermocouple itself is floating, and it simply provides closed loop temperature feedback to the PID control loop of the heater controller.

For materials characterization, we used the J.A. Woolam M-2000VI ellipsometer. It provides a 370-1690 nm measurement wavelength range.

Resistance-temperature (R-T) measurements are conducted to characterize the insulator-to-metal transition (IMT) behavior of VO_2 thin films, providing insights into their phase-transition properties and transition temperature hysteresis. The measurements are performed using a probe station together with a Keithley 2450 Sourcemeter, which enables precise resistance measurements while applying controlled voltage or current.

During the experiment, the VO_2 thin film samples are mounted on a temperature-controlled copper stage on the probe station, ensuring stable and uniform heating and cooling. Two probe needles are carefully positioned above small 2mm squares of aluminum foil placed on the VO_2 film and subsequently lowered; this establishes a two-point probe configuration with improved contact between the probe and the film's surface. For consistency, the aluminum foil squares are spaced 3mm apart, and the tips of the probes are 5mm away from one another or roughly in the center of each square.

The temperature of the sample stage is controlled using a Thorlabs TC200 Temperature Controller, which is connected directly to a flexible polyimide foil heater with a 10 kΩ

Thermistor (Thorlabs) attached to the back of a copper plate. Additionally, the copper plate is placed on ceramic spacers to provide thermal insulation from the probe station. With the software provided with the controller, the temperature of the sample stage is gradually increased from room temperature to 90 °C over the span of eight minutes, after which a constant temperature is held for five minutes. This temperature is beyond the expected IMT temperature (~68°C) and allows for time for the substrate to reach the proper temperature. The temperature is then decreased back to room temperature over ten minutes to capture both the heating and cooling branches of the transition, allowing for observing hysteresis behavior. The rate of temperature change is controlled by the computer to minimize thermal lag and ensure reliable measurements, and the resistance values were recorded using the Keithley 2450.

The substrate wafers include 4” Sapphire and Si/SiO_2 with 300 nm thick thermal oxide, purchased from University Wafer. The sapphire wafers are 650μm thick c-plane (0001) double-side-polished 4” wafer.

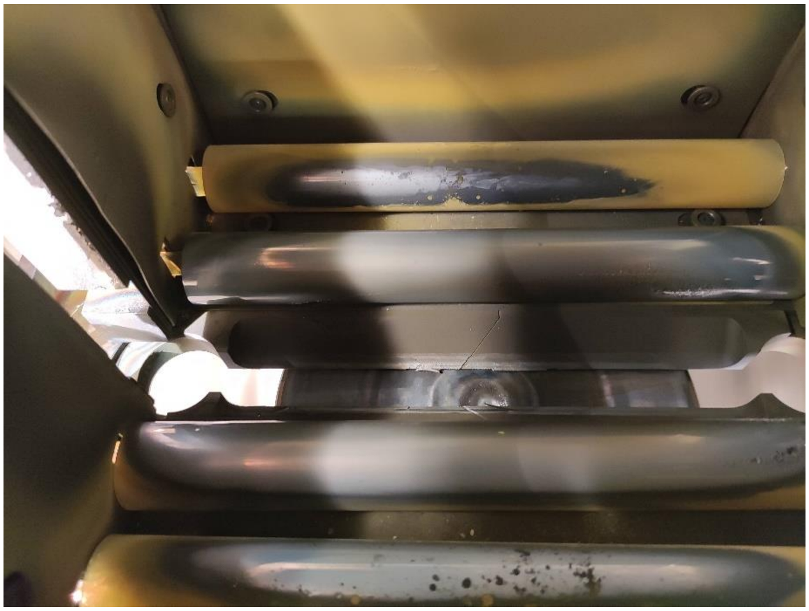

Figure S1. A picture of the inside of the PLD chamber. The target is under the laser beam slit. Four quartz tube protected heaters are above the laser beam slit.

Laser raster strategy details

In the PLD system, there are two methods to control the relative landing location of the incident laser on the target. The first is the laser beam rastering, which manipulates the kinematic mirror and provides better film uniformity and target utilization. The second is target rastering, which moves the target carousel. Target rastering is not recommended, so we do not use this method; we only use the laser beam rastering method to control the incident location.

The laser beam rastering method will guide the incident laser beam to raster along a straight path line onto the surface of the target from the left edge of the 5” (125 mm) target all the way to the right edge. The path line of the laser’s landing point does not pass through the center of the target, while it is 5mm off from the target center. Therefore, the total length of the path line is 124.6 mm. The movement of the incident point is controlled by the kinematic mirror with the software. The speed of movement on the target surface can be as slow as ~0.085 mm/s and can be as fast as ~425 mm/s.

With the developed simulation program, we proposed two different laser beam raster strategies that focus on different goals. As shown in Figure S2. On the left is a table that represents the laser spot movement strategy. The ‘location on target’ means the checkpoint on the target that the laser spot would like to move to next, and the ‘laser spot speed’ is the speed that the laser spot would like to follow to move to the checkpoint. During deposition, the laser spot will use the first speed to move to the first location, then use the second speed to move to the second location, etc. When it reaches the last row, it will start over from the first row again. With our instrument PVD products’ PLD-4000 system, the laser will pause moving at each checkpoint for about one second. This is considered during our simulation. However, similar results should be easily achieved regardless of such minor differences between different types of instruments if similar laser raster strategies are followed.

The first strategy, as shown in Figure S2, uses 20,000 pulses and will maximize the lifetime of the target since it will blast the target almost uniformly.

(a)

Location on Target(mm)	Laser Spot Speed (mm/s)
24.92	4.2671
49.84	7.1116
74.76	21.3356
99.68	7.1090
124.6	4.2671
99.68	4.26712
74.76	7.1116
49.84	21.3356
24.92	7.1116
0	4.2671

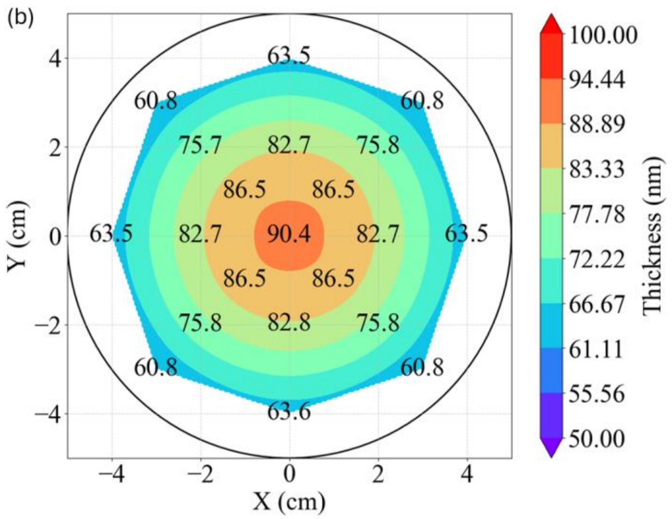

Figure S2. (a) The first strategy maximizes the target's lifetime. (b) The predicted thickness of the VO_2 thin film on the substrate with this laser raster strategy.

The second strategy uses 40,000 pulses and will deposit a uniform VO_2 thin film on the target, as shown in Figure S3. The thickness difference between the 17 points on the wafer will be < ±2%.

(a)

Location on Target(mm)	Laser spot speed (mm/s)
0	0.03413
0.4352	225.2871
13.4073	0.8705
11.6236	19.4154
0	347.3950
2.9443	338.6559
16.3942	423.8789
47.1943	426.7037
55.7883	105.4406
0.09387	426.7037

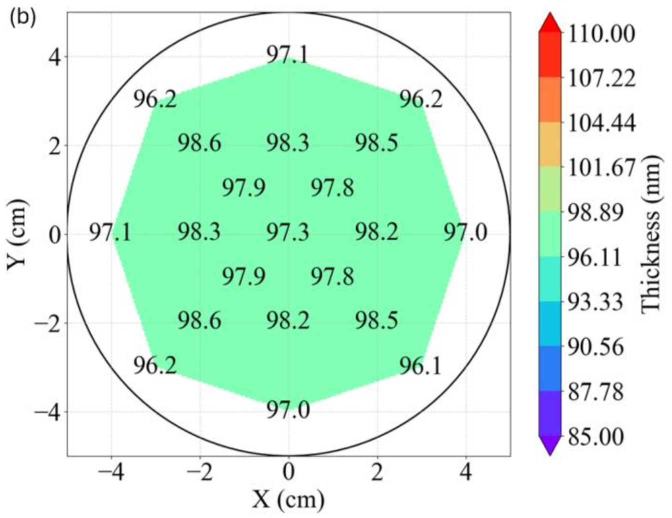

Figure S3. (a) The second strategy is to deposit a uniform VO_2 thin film. (b) The predicted thickness of the VO_2 thin film on the substrate with this laser raster strategy.

Using the second strategy with 66,000 pulses, we deposited the reported VO2 thin film on a sapphire substrate. The comparison between the experimental thickness distribution and the predicted thickness distribution is shown in Figure S4.

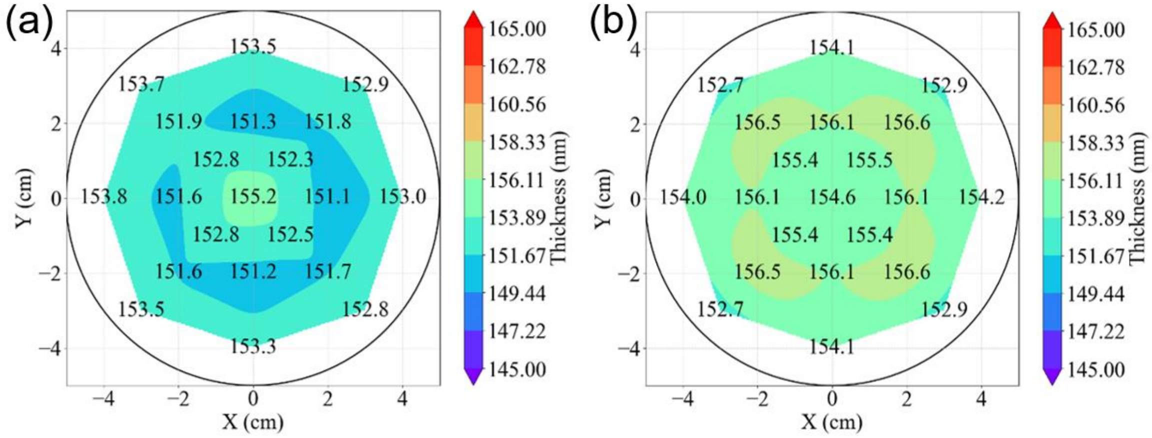

Figure S4. (a) Experimental thickness distribution of the VO_2/Sapphire wafer measured by ellipsometry. The same as Figure 2(c). (b) The model predicted the thickness distribution of the VO_2/Sapphire wafer.

Model calibration details

The d_{s0} represents the thickness of VO_2 deposited in the center of the substrate with each laser pulse hitting the target. It is directly related to the overall speed of the deposition, while we also use it to calculate the uniformity of the deposition using Scheller's model. The 'sum of error' on the left axis is calculated in the following way. First, we deposited a verification wafer and performed multi-point measurements using ellipsometry. This corresponds to the first row in Table 1 below. In Table 1, d_{s0} has the units of nm/pulse. *t(x,y)* is the thickness at the position of (x, y) cm on the wafer with a unit of nm. After measuring the verification wafer, we adjusted d_{s0} for each n value to match the predicted *t(0,0)* with the experimental value. This makes sure the thickness at the wafer center exactly matches the experimental value. Then, we used the model to calculate the thickness at the other eight locations. The total thickness difference at each location between the calculated value and the experimental value is the sum of error, measured in nm.

Table S1. Details of Model Calibration

n value	d_{s0}	*t(0,0)*	*t(2,-2)*	*t(0,-4)*	*t(-2,-2)*	*t(-4,0)*	*t(-2,2)*	*t(0,4)*	*t(2,2)*	*t(4,0)*	Sum of error
Experi-ment	N/A	82.64	66.77	57.5	66.18	51.98	66.69	58.31	66.66	48.43	N/A
n=3	0.007359723	82.64	70	59.65	70	59.63	69.98	59.63	69.99	59.64	36
n=4	0.007770395	82.64	68.59	57.31	59.58	57.3	68.56	57.29	58.57	57.31	33.79
n=5	0.008181293	82.64	67.28	55.22	67.28	55.21	67.26	55.2	67.27	55.22	18.2
n=6	0.008591397	82.64	66.07	53.34	66.06	53.32	66.05	53.32	66.06	53.34	17.46
n=7	0.008999366	82.64	64.93	51.63	64.93	51.61	64.91	51.6	64.92	51.62	22.75
n=8	0.009405686	82.64	63.87	50.07	63.86	50.05	63.84	50.04	63.85	50.06	30.14
n=9	0.009808296	82.64	62.85	48.63	62.84	48.61	62.82	48.6	62.83	48.63	37.11

Deposition conditions details

We investigate the effect of O_2 pressure during the deposition of VO_2 thin films. These samples are deposited with the same 600 °C temperature, 10 sccm O_2 flow rate, and 90 mm target to substrate distance. 20 minutes of annealing at the same temperature in vacuum is carried out in every deposition. Figure S5 shows the SEM images of $VO_2/SiO_2/Si$ samples deposited with different O_2 pressures.

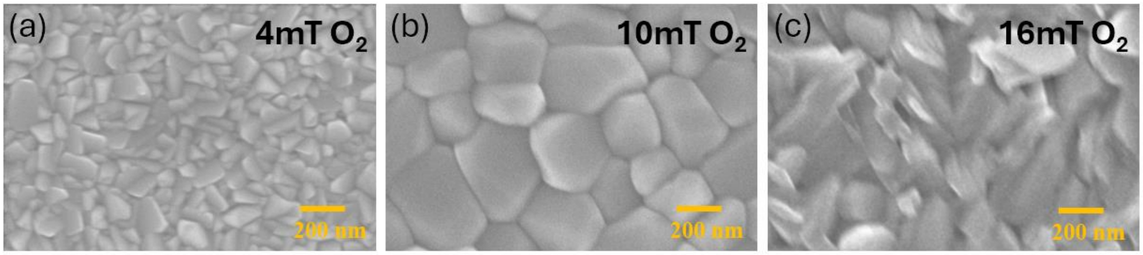

Figure S5. SEM images of the VO_2 thin film deposited on SiO_2/Si substrates with different O_2 pressures. (a) with 4 mTorr O_2. (b) with 10 mTorr O_2. (c) with 16 mTorr O_2.

We also investigate the effect of deposition temperature on the morphology of the VO_2 thin film. These samples are deposited with the same 10 mTorr O_2 pressure, 10 sccm O_2 flow rate, and 90 mm target to substrate distance.

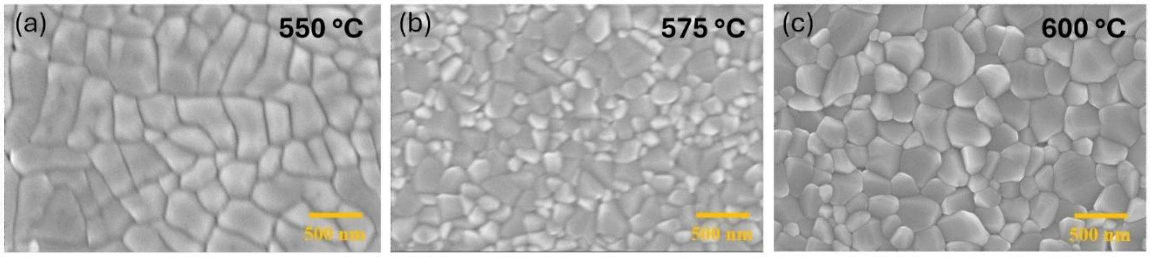

Figure S6. SEM images of the VO_2 thin film deposited on SiO_2/Si substrates with different temperatures. (a) at 550 °C. (b) at 575 °C. (c) at 600 °C.

Ellipsometry characterization details

We used ellipsometry to characterize our samples. After the sample wafer was fabricated, we performed a 21-point scan on the sample wafer. The 21 points' locations are (0, 0), (1, 1), (4, 0), …, etc., with the unit of centimeters. This will provide insight into the properties of the VO_2 thin film across different locations on the wafer.

The ellipsometer provides a 370-1690 nm measurement wavelength range and multiple incident angle options. We used 65° as the incident angle during the measurement. Once the measurement was done, we carried out the standard data processing and fitting with the data. The Cauchy model is used with a fitting wavelength range from 600 nm to 1690 nm. We fit all the optical parameters of the thin film, including its thickness and roughness. The fitting turns out very good, and all the MSE values are less than 15. Then, with the fitting parameters, we could generate the refractive index (n) and extinction coefficient (k) plots of the VO_2 thin film.

Atomic force microscopy (AFM) characterization details

We also used the atomic force microscope (AFM) to characterize the VO_2 thin film on sapphire. The AFM image is shown in Figure S7. The root mean square (RMS) roughness (Sq) is 29.4 nm, almost the same as the fitted roughness from the ellipsometer.

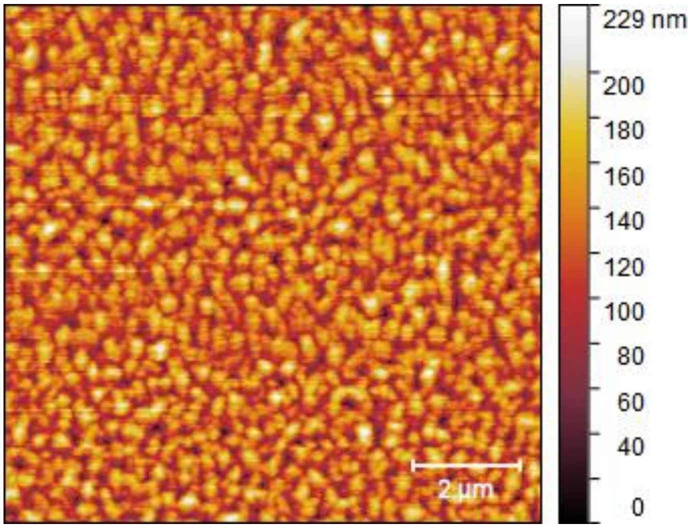

Figure S7. AFM image of the VO_2/Sapphire sample.

Historical resistance vs. temperature data

Here, we listed the measured resistance vs. temperature results of our VO_2 samples with the sample date. Measurements were taken within two days of the listed deposition date.

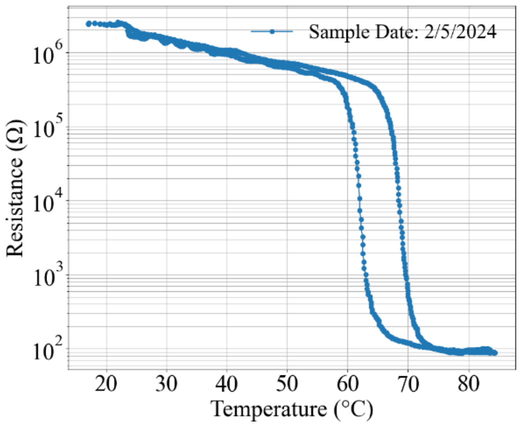

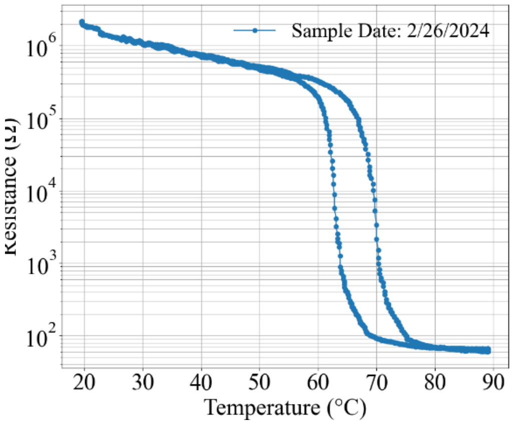

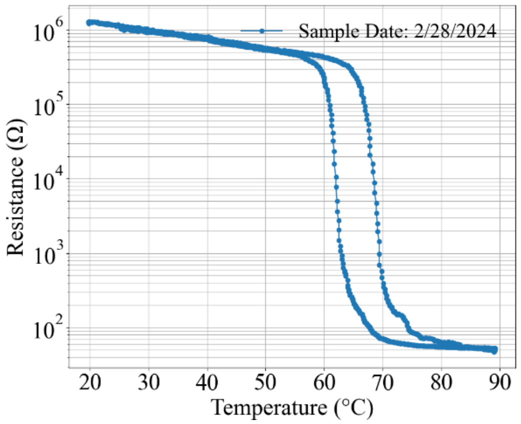

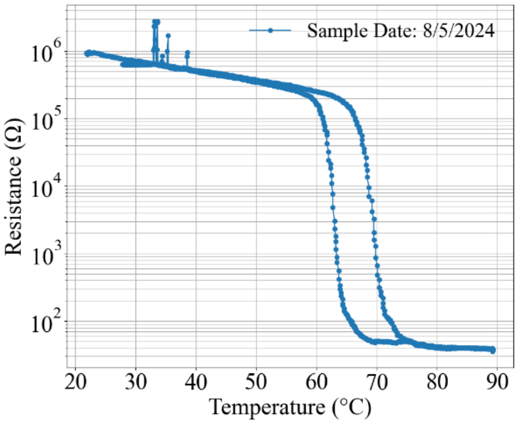

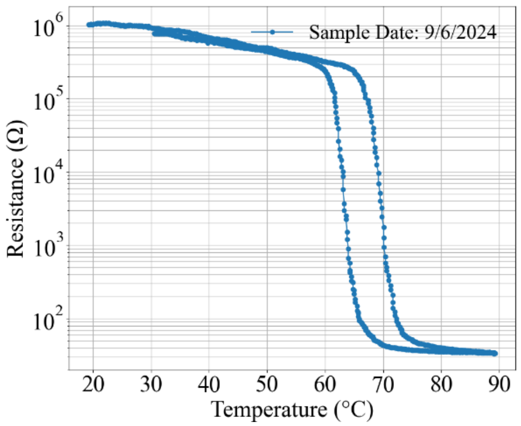

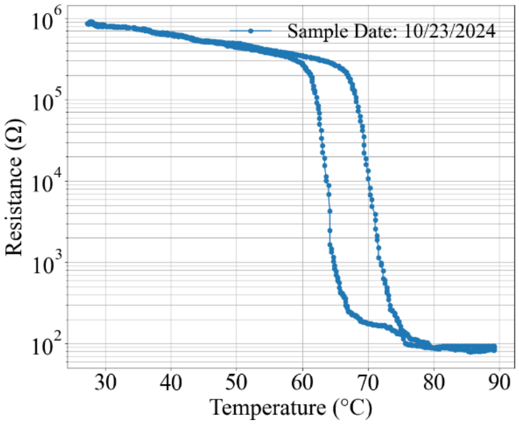

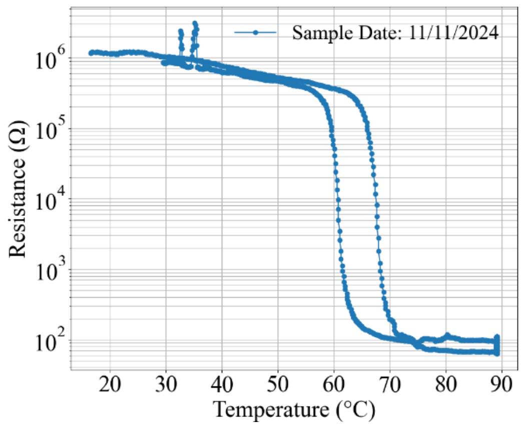

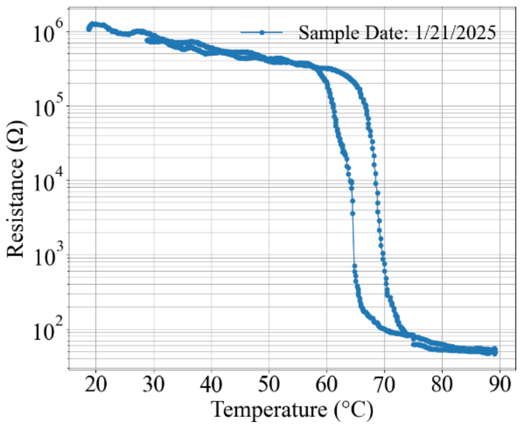

Note:

The sample fabricated on 2/5/2024 was the sample used in our paper "Switchable Terahertz Beam Steering with Near-Perfect Ordinary Transmission. Advanced Photonics Research, 2500073 (2025). https://doi.org/10.1002/adpr.202500073."

Comparison with Previous VO_2 Works

We also compared the quality of our deposited VO_2 thin films with previous works; the comparison of the order of magnitude change in resistance during phase transition is shown in Table S2. Among all results, our work shows the highest resistance change during the insulator to metallic transition (IMT) when compared with other works using different synthesis methods.

Table S2. Summary of reported magnitude of resistance changes of VO_2 thin films across the phase transition

Name	Synthesis Methods	Order of Magnitude Change in Resistance During IMT (30°C-90°C)
This work	PLD	4.4

Growth and phase transition characteristics of pure M-phase VO_2 epitaxial film prepared by oxide molecular beam epitaxy[2]	MBE	4.0
Transport properties of ultra-thin VO_2 films on (001) TiO2 grown by reactive molecular-beam epitaxy[3]	MBE	3.7
Thickness-modulated metal–insulator transition of VO_2 film grown on sapphire substrate by MBE[4]	MBE	4.0
Improved metal-insulator-transition characteristics of ultrathin VO_2 epitaxial films by optimized surface preparation of rutile TiO2 substrates[5]	PLD	3.5
Synthesis of phase pure vanadium dioxide (VO_2) thin film by reactive pulsed laser deposition[6]	PLD	3.0
Phase changeable vanadium dioxide (VO_2) thin films grown from vanadium pentoxide (V_2O_5) using femtosecond pulsed laser deposition[7]	PLD	3.8
rf-microwave switches based on reversible semiconductor metal transition of VO_2 thin films synthesized by pulsed laser deposition[8]	PLD	3.0
Insulator–metal transition in substrate-independent VO_2 thin film for phase-change devices[9]	Sputtering	3.4
VO_2 thin films with low phase transition temperature grown on ZnO/glass by	Sputtering	3.0

applying substrate DC bias at low temperature of 250 °C[10]		
Smart Windows with a VO_2 Thin Film as a Conductive Layer for Efficient and Independent Dual-Band Modulation[11]	Sputtering	3.2
MOCVD growth and characterization of vanadium dioxide films[12]	CVD	4.0
Defect engineering of VO_2 thin films synthesized by Chemical Vapor Deposition[13]	CVD	2.8
Direct synthesis of high-performance thermal sensitive VO_2(B) thin film by chemical vapor deposition for using in uncooled infrared detectors[14]	CVD	<1

Reference

(1) PVD Products, I. *PLD-4000 Deposition System Operation and Service*; 2019/4.

(2) Fan, L. L.; Chen, S.; Wu, Y. F.; Chen, F. H.; Chu, W. S.; Chen, X.; Zou, C. W.; Wu, Z. Y. Growth and phase transition characteristics of pure M-phase VO2 epitaxial film prepared by oxide molecular beam epitaxy. *Applied Physics Letters* **2013**, *103* (13), 131914. DOI: 10.1063/1.4823511 (accessed 2/7/2025).

(3) Paik, H.; Moyer, J. A.; Spila, T.; Tashman, J. W.; Mundy, J. A.; Freeman, E.; Shukla, N.; Lapano, J. M.; Engel-Herbert, R.; Zander, W.; et al. Transport properties of ultra-thin VO2 films on (001) TiO2 grown by reactive molecular-beam epitaxy. *Applied Physics Letters* **2015**, *107* (16), 163101. DOI: 10.1063/1.4932123 (accessed 2/7/2025).

(4) Bian, J.; Wang, M.; Sun, H.; Liu, H.; Li, X.; Luo, Y.; Zhang, Y. Thickness-modulated metal–insulator transition of VO2 film grown on sapphire substrate by MBE. *Journal of Materials Science* **2016**, *51* (13), 6149-6155. DOI: 10.1007/s10853-016-9863-1.

(5) Martens, K.; Aetukuri, N.; Jeong, J.; Samant, M. G.; Parkin, S. S. P. Improved metal-insulator-transition characteristics of ultrathin VO2 epitaxial films by optimized surface preparation of rutile TiO2 substrates. *Applied Physics Letters* **2014**, *104* (8), 081918. DOI: 10.1063/1.4866037 (accessed 3/12/2025).
(6) Bhardwaj, D.; Goswami, A.; Umarji, A. M. Synthesis of phase pure vanadium dioxide (VO2) thin film by reactive pulsed laser deposition. *Journal of Applied Physics* **2018**, *124* (13). DOI: 10.1063/1.5046455.
(7) Kumi-Barimah, E.; Anagnostou, D. E.; Jose, G. Phase changeable vanadium dioxide (VO2) thin films grown from vanadium pentoxide (V2O5) using femtosecond pulsed laser deposition. *AIP Advances* **2020**, *10* (6), 065225. DOI: 10.1063/5.0010157.
(8) Dumas-Bouchiat;, F.; Champeaux;, C.; Catherinot;, A.; Crunteanu;, A.; Blondy, P. rf-microwave switches based on reversible semiconductor-metal transition of VO2 thin films synthesized by pulsed-laser deposition. *APPLIED PHYSICS LETTERS* **2007**, *91* (22), 223505. DOI: 10.1063/1.2815927.
(9) Taha, M.; Walia, S.; Ahmed, T.; Headland, D.; Withayachumnankul, W.; Sriram, S.; Bhaskaran, M. Insulator-metal transition in substrate-independent VO(2) thin film for phase-change devices. *Sci Rep* **2017**, *7* (1), 17899. DOI: 10.1038/s41598-017-17937-3 From NLM PubMed-not-MEDLINE.
(10) Zhu, M.; Qi, H.; Li, C.; Wang, B.; Wang, H.; Guan, T.; Zhang, D. VO2 thin films with low phase transition temperature grown on ZnO/glass by applying substrate DC bias at low temperature of 250 °C. *Applied Surface Science* **2018**, *453*, 23-30. DOI: 10.1016/j.apsusc.2018.05.089.
(11) Sang, J.; Zhu, W.; Feng, Y.; Liu, Y.; Shang, J.; Sun, J.; Guo, L.; Zhang, Y.; Zhao, S.; Chigrinov, V.; et al. Smart Windows with a VO2 Thin Film as a Conductive Layer for Efficient and Independent Dual-Band Modulation. *ACS Applied Electronic Materials* **2021**, *3* (11), 4882-4890. DOI: 10.1021/acsaelm.1c00728.
(12) Yakovkina, L. V.; Mutilin, S. V.; Prinz, V. Y.; Smirnova, T. P.; Shayapov, V. R.; Korol'kov, I. V.; Maksimovsky, E. A.; Volchok, N. D. MOCVD growth and

characterization of vanadium dioxide films. *Journal of Materials Science* **2016**, *52* (7), 4061-4069. DOI: 10.1007/s10853-016-0669-y.
(13) Rajeswaran, B.; Umarji, A. M. Defect engineering of VO2 thin films synthesized by Chemical Vapor Deposition. *Materials Chemistry and Physics* **2020**, *245*, 122230. DOI: 10.1016/j.matchemphys.2019.122230.
(14) Guo, B.; Wan, D.; Ishaq, A.; Luo, H.; Gao, Y. Direct synthesis of high-performance thermal sensitive VO2(B) thin film by chemical vapor deposition for using in uncooled infrared detectors. *Journal of Alloys and Compounds* **2017**, *715*, 129-136. DOI: 10.1016/j.jallcom.2017.04.304.
